# Flow Diversion for the Management of Ruptured Intracranial Arterial Infudibular Dilatation: Proof of Principle and Therapeutic Protocol

**DOI:** 10.3389/fneur.2022.913879

**Published:** 2022-05-24

**Authors:** Svetozar Matanov, Kristina Sirakova, Kalina Chupetlovksa, Marin Penkov, Dimitar Monov, Martin Krupev, Krasimir Minkin, Kristian Ninov, Vasil Karakostov, Stanimir Sirakov

**Affiliations:** ^1^University Hospital St. Ivan Rilski, Sofia, Bulgaria; ^2^Alexandrovska Hospital, Sofia, Bulgaria

**Keywords:** infundibular dilatation, posterior communicating arteries, subarachnoid hemorrhage, flow diverter, anterior choroidal artery

## Abstract

Thought to be benign anatomical variants, cerebral infundibular dilatations (ID) are most commonly encountered at the junction of the internal carotid artery (ICA) and the posterior communicating artery (PcomA). The true nature of this entity remains controversial, as some literature reports suggest they should be considered preaneurysmal lesions and a potential source of devastating subarachnoid hemorrhage. This report describes cases of presumably ruptured IDs and their therapeutic endovascular management. We retrospectively reviewed and analyzed patients with isolated subarachnoid hemorrhage (SAH) where the only potential cause was ruptured cerebral IDs, treated or not, between January 2012 and June 2021. Morphological and radiological features, treatment and procedural considerations, clinical and angiographic outcomes were also reviewed. Natural history of the ID is poorly understood, and its relation to SAH remains controversial. Ruptured cerebral IDs can be the suspected cause of bleeding if no other vascular lesion is present during multimodal examinations. Endovascular flow diversion stenting is safe and effective for the proper treatment of ruptured IDs. Pending further validations with longitudinal data are needed to legitimate the natural course of these mysterious lesions.

## Introduction

**Figure 1:**
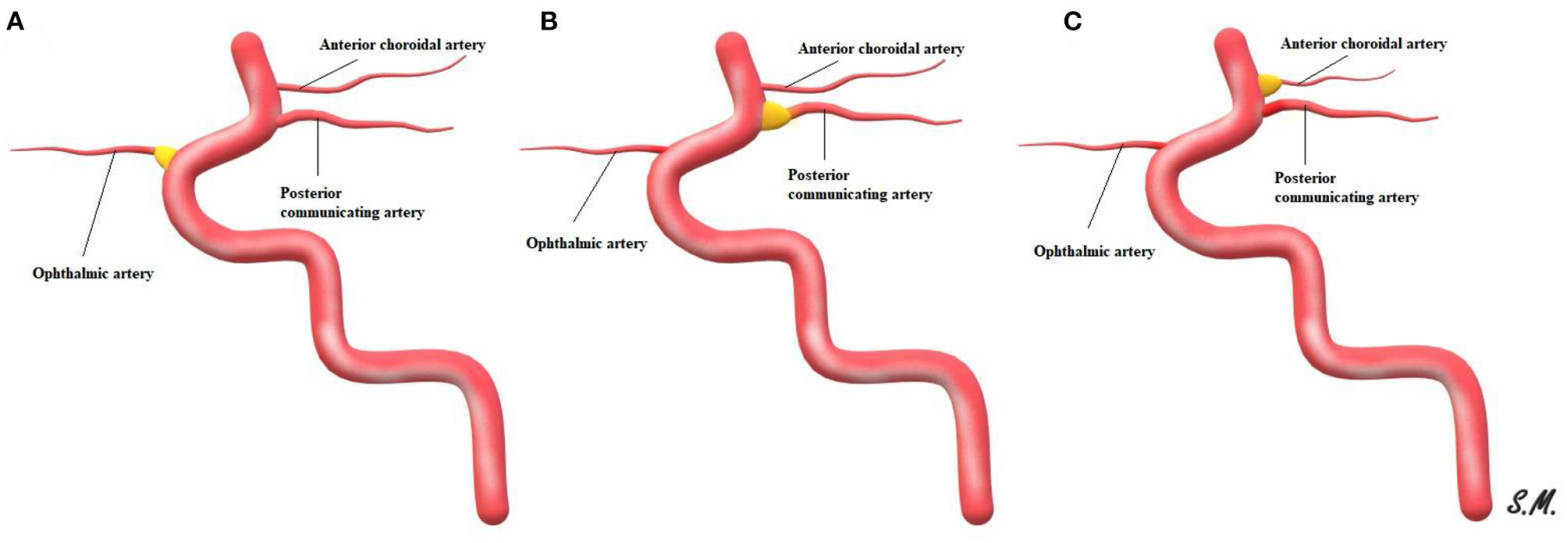
Schematic representation of ID with the adjacent origin of an intracranial artery, ophthalmic artery ID **(A)**, posterior communicating artery ID **(B)** and ID at anterior choroidal artery **(C)**.

The clinical significance of an infundibular dilatation (ID) at the origin of an intracranial artery remains a matter of debate. Those junctional dilatations have been postulated to have specific radiological characteristics with qualitative parameters including a maximum diameter of 3 mm, teardrop bulging or funnel-like shapes, and a lack of an aneurysmal-like neck (1). An infundibulum usually can be seen at branching sites within the intracranial circulation, typically at the internal carotid artery (ICA) and the posterior communicating artery orifice (2). However, IDs can less frequently occur at the level of the anterior choroidal artery, ophthalmic artery, anterior communicating artery, or superior cerebellar artery, or across the middle cerebral artery (Figure 1) (3, 4).

Although little is known regarding the actual natural courses of these entities, intracranial arterial infundibulums are commonly encountered during neurovascular clinical examinations (5). The reported incidence varies between 2.2 and 24.6% at the population level, and these entities may be misdiagnosed as aneurysms on non-invasive imaging (6, 7). IDs are commonly accepted to be benign lesions or normal anatomic variations whose clinical significance is unclear or non-existent (8). However, multiple reports have described ruptures with clinical sequelae and aneurysmal transformations of an ID (9–12). A diagnostic and therapeutic dilemma may occur only after a junctional dilatation is identified in the setting of unexplained subarachnoid hemorrhage (SAH) (13). The available literature is scarce and insufficiently consistent to entirely resolve the clinical uncertainties of IDs. Herein, we aim to emphasize and report the experience of a moderate-volume single center regarding the diagnosis and treatment management of ruptured IDs in the setting of otherwise unexplained SAH.

## Methods

### Patients

An institutional review board-approved retrospective review was performed to identify and analyze patients with isolated and non-aneurysmal SAH. The only objective cause was ruptured cranial IDs, either treated or untreated, between January 2012 and June 2021. A search of the institutional medical record database revealed eight patients with IDs as a potential source of SAH. Among them, we treated six patients whose cases had not been previously published or reported.

IDs were defined as lesions meeting the following explicit morphological criteria: symmetrical cone-like outpouching at the level of the arterial ostium, with no distinct neck and a maximum diameter <3 mm. SAH due to ruptured ID was considered a diagnosis if no other vascular lesions were present during multimodal radiological examinations and delayed (7 days after onset) angiographic imaging. Three-dimensional rotational angiography (3DRA) was used to rule out blister aneurysms or dissecting aneurysms involving the PcomA origin that might mimic an ID.

The infundibulum's geometric ratios and morphological characteristics, and the involved parent artery were assessed during diagnosis, treatment, and radiological follow-up.

The patients' demographics, clinical and neurological status, and radiological records were obtained and reviewed. The clinical outcomes were measured and evaluated with the modified Rankin Scale at discharge. The parents' arterial vascular remodeling and ID occlusion were assessed according to the O'Kelly-Marotta scale during the endovascular procedure and 3–12 months after flow diversion (14).

### Treatment Considerations

According to our local institutional policy for all patients diagnosed with an acute SAH due to a possible ID rupture, all cases were discussed in neurovascular multidisciplinary team meetings. Concerns regarding the diagnosis itself, treatment indications, and modalities were fundamental aspects of each case analysis. The treatment decisions were based on all the aforementioned medical criteria, including the anticipated rebleeding prevention.

In general, the medical management of these patients did not significantly diverge from commonly accepted therapeutic protocols (15, 16). The only protocol deviation was the mandatory antiplatelet therapy on the conducted flow diverter stent (FD) implantation. The deployment of the flow modulation stent was aimed to fully cover IDs and the ostium of the involved parent artery.

### Management of Antiplatelet Therapy

All patients undergoing endovascular intervention received either a loading dose (per os) of aspirin (300 mg) and prasugrel (40 mg) 7 h before the procedure or an intraprocedural bolus dosage of glycoprotein IIb/IIIa inhibitor. In the post-treatment phase, a standard daily dose of 1 × 10 mg of prasugrel and 100 mg of aspirin were maintained for at least 6 months. Adequate therapy response was measured with VerifyNow assays (Accumetrics, San Diego, CA, USA) with a desired PRU < 120.

## Results

### Patients' Baseline and Clinical Characteristics

The demographics and clinical details of the reviewed patients are summarized and presented in Table 1. We identified eight patients (six of whom were women), whose mean age was 44.5 years (range 19–60). Most IDs were located at the level of the PcomA (*n* = 7), and only one was located on the arterial orifice of the ophthalmic artery (OA). All patients reported in our series presented with radiologically confirmed acute SAH. Only one (12.5%) patient experienced SAH after a re-rupture earlier in life. Pretreatment Hunt and Hess (H&H) grade I and II were observed in five (62.5%) patients. However, two (25%) patients experienced severe and diffuse SAH resulting in H&H grade III, and one had an H&H score of IV.

**Table 1:** Patients' baseline characteristics.

**Case**	**Age**	**Sex**	**Location**	**Hunt-Hess**	**Fisher**	**ID size**	**Similar previous**	**Confirmed on initial**	**EVT treatment and**	**Clinical outcome**	**Clinical outcome**
				**grade**	**scale**	**and features**	**symptoms**	**imaging (modality)**	**used stent**	**at discharge (mRS)**	**at 12-month FU (mRS)**
1	40s	F	L PcomA	Grade I	II	2.7 mm cone	No	CTA/DSA	FD (p64)	0	0
2	40s	F	L PcomA	Grade II	II	<3 mm cone	No	DSA/3D RA	FD (PED-S)	0	0
3	60s	M	L PcomA	Grade II	III	<3 mm cone	No	DSA/3D RA	FD (p64)	0	0
4	50s	F	L PcomA	Grade I	II	<3 mm cone	No	DSA/3D RA	Refused	N/A	N/A
5	40s	M	R PcomA	Grade III	III	<3 mm cone	No	CTA/DSA	Refused	N/A	N/A
6	40s	F	R PcomA	Grade IV	IV	<3 mm cone	No	CTA/DSA	FD (PED-S)	1	0
7	40s	F	L PcomA	Grade III	III	<3 mm bleb (1 mm)	Yes 5 years ago	CTA/DSA	Failed coil/FD(p64)	1	0
8	≥19	F	L OA	Grade I	III	2.5 mm cone	No	DSA/3D RA	FD (p64)	0	0

*SAH, subarachnoid hemorrhage; F, female; FU, follow up; M, male; mRS, modified Rankin Scale; NA, not applicable; L/R, left or right; PcomA, posterior communicating artery; OA, ophthalmic artery; FD, flow diverter stent; CTA, computed tomography angiography; DSA, digital subtraction angiography; 3D RA, three- dimensional rotational angiography; p46, the p64 Flow diverter stent; PED-S, Pipeline Embolization Device with Shield Technology*.

In all cases, the initial computed tomography angiography (CTA) did not indicate that the presence of an aneurysm or any other vascular lesion was responsible for the SAH. However, in four cases, unilateral PcomA IDs were noted. In those cases, the SAH distribution from the non-contrast-enhanced CT scan was located predominantly near the anatomical location of the observed PcomA IDs into the origin of the ipsilateral suprasellar cistern and Sylvian fissure. One patient, patient No. 7, had a prior history of aneurysmal negative SAH 5 years prior.

The four IDs detected in the CTA were verified through subsequent digitally subtracted angiography (DSA). In general, DSA and preoperative 3DRA detected all IDs and indicated an absence of any other vascular pathology associated with the SAH. Delayed DSA examinations 7 days after onset confirmed the above findings in all eight patients. Endovascular therapy (EVT) of the ruptured ID was performed in six (75%) patients. Two patients (25%) rejected the therapeutic intervention because of their individual beliefs.

### Treatment Modality and Feasibility

The technical, procedural, and clinical results are summarized in Table 2.

**Table 2:** Procedural, clinical, and follow-up angiographic results after the endovascular intervention.

**Patient**	**Time to treatment**	**PRU**	**EVT**	**Clinical sequelae**	**Observed rebleeding**	**First angiographic**	**First clinical**
**number**	**since ictus**	**results**	**result**	**of complications**	**or retreatment**	**follow-up, ID occlusion—OKM scale**	**follow-up**
1	12 days	28 PRU	Stagnation	None	No	3 months OKM D	No symp
2	11 days	17 PRU	Stagnation	None	No	3 months OKM D	No symp
3	7 days	56 PRU	No filling	None	No	4 months OKM D	No symp
4	7 days	44 PRU	Stagnation	None	No	4 months OKM D	No symp
5	10 days	35 PRU	No filling	None	No	4 months OKM D	No symp
6	7 days	25 PRU	Stagnation	None	No	3 months OKM D	Headaches

*EVT, endovascular therapy; N/A, not available; PRU, platelet Response Unit; ID, infundibular dilatation; OKM, O'kelly Marotta Scale*.

Of the eight patients with SAH due to a ruptured ID, six underwent EVT in the acute phase of SAH. For the patients undergoing EVT, treatment of the ruptured ID was performed *via* implantation of an FD across the documented focal lesion. In all patients, only one FD was used; of the FDs used, two (33.3%) had additional antithrombotic surface coating. In all procedures, the attempted implantation of the FD was feasible. No unexpected procedure-related complications were encountered. No cases of recurrent bleeding resulted from the endovascular intervention or the mandatory antiplatelet medication. SAH related complications were observed, and all six patients demonstrated favorable outcomes, with modified Rankin Scale scores of 0 or 1 at discharge.

### Angiographic and Clinical Follow-Up

At least two cross-sectional radiological and clinical follow-ups were available for all patients who underwent EVT. The two patients who refused treatment were lost to follow-up after the initial presentation and discharge. The initial angiographic follow-up was conducted after a median of 3 months (range 3–4 months) after the intervention. No patients reported any post-treatment clinical and neurological symptoms associated with ID rerupture during the follow-up examinations. No mortality or morbidity was observed in this group.

### Illustrative Cases

#### Case 1

**Figure 2:**
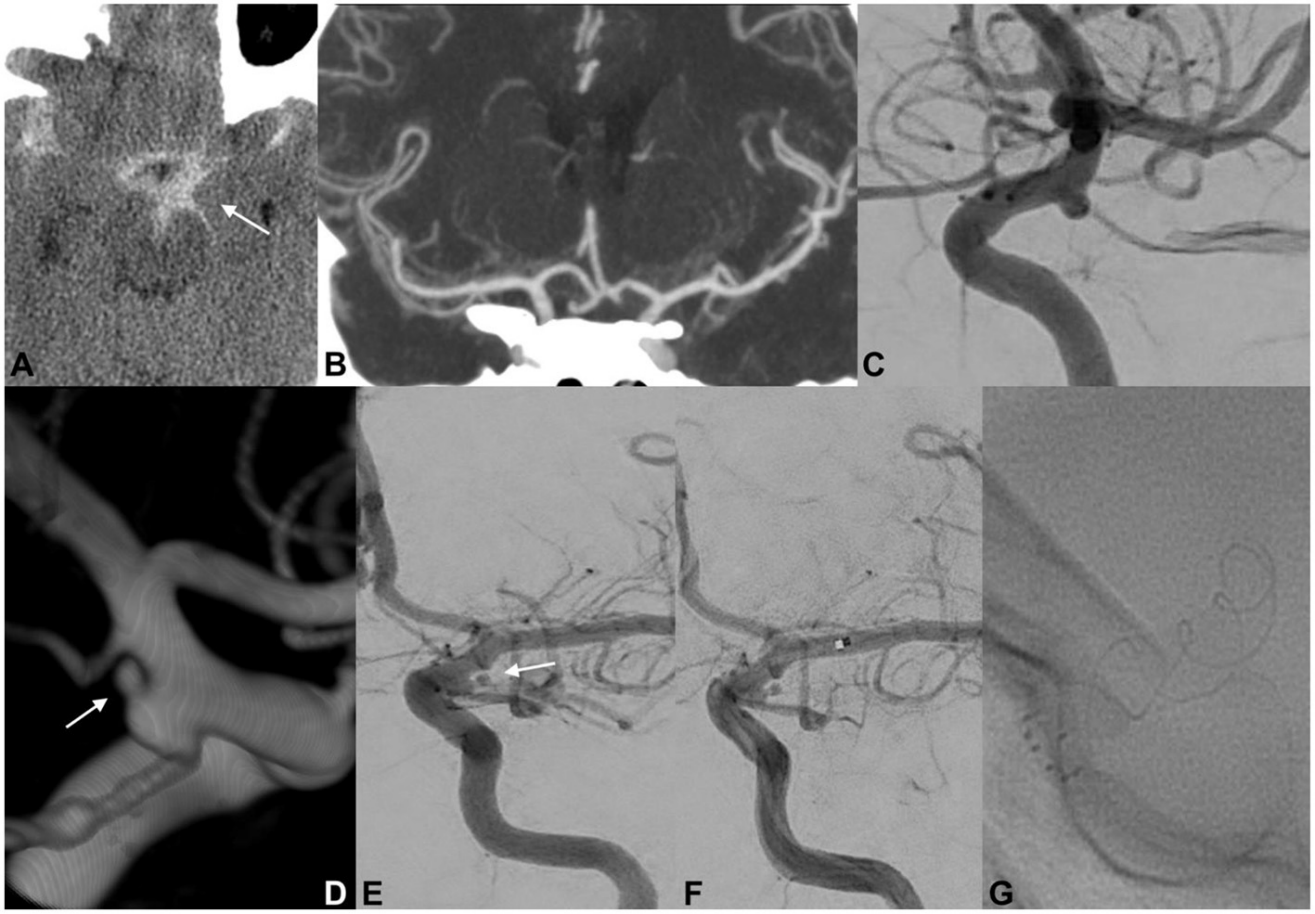
EVT of a ruptured PcomA ID. An initial CT scan demonstrated the presence of SAH predominantly distributed across the left basal cisterns (**A**; white arrow). Cranial CTA did not document any cerebrovascular findings that could have caused the SAH **(B)**. At 6 h after onset, catheter angiography confirmed the absence of a ruptured aneurysm but revealed a typical ID at the origin of the left PcomA **(C)**. Delayed 3DRA/DSA (**D,E**; white arrows) 7 days after onset identified a possible rupture bleb on the lateral side of the left PcomA ID. Endovascular coil embolization of the ruptured bleb was unsuccessful, and a p64 FD was carefully deployed across the C7 segment of the left ICA **(F,G)**. Contrast stagnation inside the ruptured ID bleb was seen on the delayed angiographic phase (not provided).

A 46-year-old woman was admitted to another hospital for a sudden thunderclap headache (Figure 2). CT revealed diffuse SAH with increased blood collection in the left suprasellar cistern and left-sided Sylvian fissure. Subsequent CTA did not confirm the presence of any cerebrovascular findings that could be responsible for the acute SAH. DSA did not detect any intracranial aneurysms but revealed an ID at the origin of the left PcomA. The delayed 3DRA/DSA 7 days after onset identified a possible rupture bleb on the lateral side of the left PcomA ID. The patient had a history of an identical episode occurring 5 years earlier, but the radiological examinations did not confirm any intracranial hemorrhage. The patient's medical records included trombangiitis obliterans (Buerger disease), idiopathic thrombocytopenic purpura, and uncontrolled hypertonia. On the basis of the above findings, we concluded that the tiny bleb on the PcomA ID was responsible for the intracranial hemorrhage. Endovascular coil embolization of the ruptured infundibular bleb was performed but was unsuccessful. Coiling of the ruptured dilatation was not possible because the coils protruded toward the parent artery's lumen. Bailout flow diversion was considered appropriate for this case. The patient received a bolus dosage of glycoprotein IIb/IIIa–tirofiban and received a p64 (phenox, Bochum, Germany) FD. Contrast stagnation inside the ruptured bleb was observed on the final angiograph. The patient was discharged after 14 days with no new neurological deficits detected.

#### Case 2

**Figure 3:**
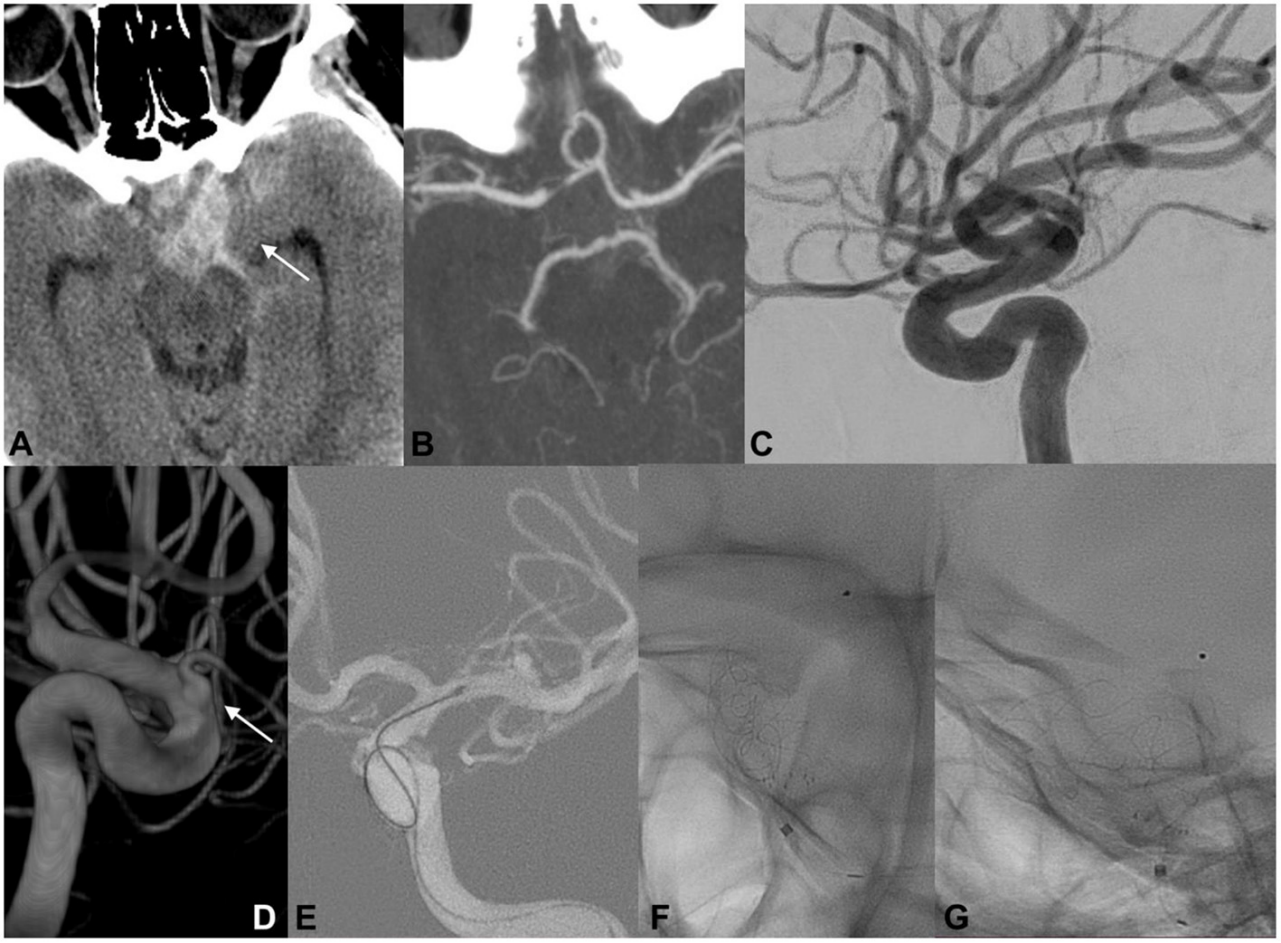
EVT of ruptured OA ID in a young woman. The initial radiological work-up indicated intracranial hemorrhage, suggesting the rupture of an intracranial aneurysm (**A**; white arrow). Cranial CTA and initial DSA did not reveal any cerebrovascular pathologies that could be associated with the hemorrhage **(B,C)**. Three-dimensional rotational angiography demonstrated the presence of an ID at the level of the orifice of the left ophthalmic artery (**D**; white arrow). Endovascular FD deployment was performed with complete coverage of the OA infundibular orifice **(E–G)**.

A 19-year-old woman was found unconscious at home (Figure 3). Her last memory was of an extreme burning sensation engulfing her neck and scalp. CT revealed a thin SAH mainly distributed in the left suprasellar cistern and left hemispheral subarachnoid spaces. CT cranial angiography and initial DSA did not indicate any aneurysmal dilatations or other vascular findings. Subsequently, 3DRA revealed the presence of an ID at the level of the orifice of the left ophthalmic artery. The secondary delayed DSA indicated the presence of a focal irregularity adjacent to the OA ID. The patient repeatedly reported having observed increasing flashes of light and bright spots in her left eye during this period. A ruptured OA ID was hypothesized to have been responsible for the cerebral hemorrhage, and the patient was scheduled for EVT with an FD stent. The patient was premedicated with a loading dose of DAP, and the implantation of the endoluminal flow modulation device was performed uneventfully. The patient was discharged without any related procedural complications. Follow-up angiography demonstrated no infundibular widening and the complete preservation of the OA.

#### Case 3

**Figure 4:**
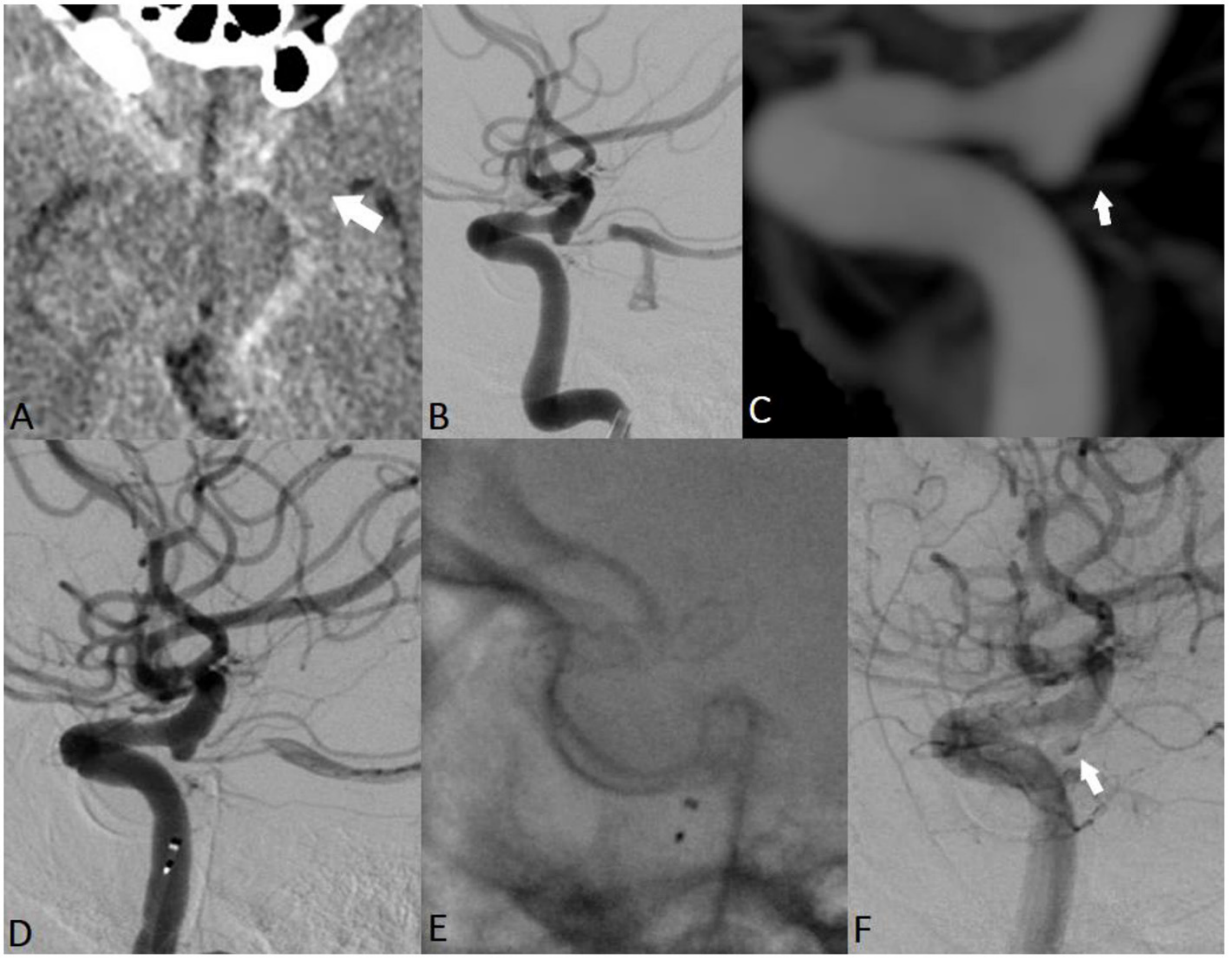
EVT of a ruptured PcomA ID in a 60-year-old man. Non-contrast cranial CT revealed a thin hemorrhage in the chiasmatic, interpeduncular, and left crural cisterns (**A**; white arrow). Subsequent repeated radiological examinations did not reveal any cerebrovascular pathology but confirmed the presence of an ID at the level of the left PcomA (**B,C**; white arrow). The assumed rupture site was the observed ID, and the patient received EVT **(D,E)**. Noticeable contrast stagnation was observed in the PcomA ID after stent implantation **(F)**.

A 60-year-old man was admitted to the hospital with severe headache, nausea, vomiting, and prominent neck stiffness (Figure 4). He reported that the headache intensity had worsened in the prior 7 days and did not respond to analgesics. CT demonstrated the presence of a diffuse SAH with a focal distribution mainly into the chiasmatic, interpeduncular, and left crural cisterns. CTA did not reveal any cerebrovascular pathology but confirmed the presence of an ID at the level of the left PcomA. The patient underwent catheter angiography, which yielded similar observations to those of the initial and delayed DSA examinations. Considering the above findings, we assumed that the most probable cause of the SAH was the rupture of the PcomA ID on the left. A loading dose of DAP was administered, and the patient underwent uneventful FD placement across the C7 segment of the left ICA. Noticeable contrast stagnation was found in the PcomA ID after stent implantation. The patient was discharged after 2 weeks of conservative management with no neurological deficits. Both mid- and long-term follow-up radiological examinations confirmed the complete remodeling of the PcomA with total obliteration of the treated ID.

## Discussion

### Pathogenesis and Histological Considerations

Arterial IDs are benign lesions commonly believed to be present in as many as one-quarter of cerebral angiograms (7, 13). Although these findings are considered normal vascular variants, whether they should be considered aneurysmal precursors, and candidates for clinical surveillance and potential treatment, remains a matter of debate (8, 17). We cannot fully determine whether this paradoxical understanding of IDs was initially derived from a divergence among published histological findings. Histologically, one of the first assumptions promoted by Hassler and Saltzmann was that cerebral infundibulum might resemble an aneurysm (18). Degenerative changes and arterial wall alterations were also noted in later studies histologically examining ID samples (19, 20). However, neither abnormalities nor structural arterial defects were observed in patients with junctional dilatations in the histological studies performed by Epstein et al. (21), who proposed that these conical dilatations might merely be developmental vessel remnants with non-conditional clinical significance.

Regardless of whether IDs exhibit degenerative changes in the internal elastic lamina, the lytic process of elastase has been demonstrated to play a role in the degenerative changes directly leading to aneurismal development (22). Small defects and structural anomalies within the internal elastic lamina have been documented in post-mortem analyses of ruptured ID (23). These biological events might result from focal disruption of the homeostatic balance in the affected arterial vessel wall, as a result of increased circumferential wall stress (24). Coupe et al. (25) have reported a surgically treated case of a ruptured ID with a macroscopically evident perforation site, thus suggesting that the ID bleeding might have resulted from wall weakness without prerequired aneurysmal transformation. The very geometrical definition of arterial infundibulum or funnel-shaped widenings at the branching sites of major cerebral arteries may lead to local fluid dynamics that result in further ID dilatation (26). Marshman et al. (27) have suggested that these hydraulic distentions, conditioned by Bernoulli's principle, might be directly associated with later aneurysmal transformation of an ID.

### Clinical Significance

Although arterial IDs are present in up to 25% of cranial angiographs, fully distinguishing them from their aneurysmal counterparts remains difficult. Currently, non-invasive radiological imaging modalities, i.e., CTA and magnetic resonance angiography, have become the primary screening techniques used to detect, differentiate, and evaluate cerebral aneurysms. Superioinferior projections of cerebral CTA can sometimes aid in revealing the horizontal direction of the infundibulum. CTA has a considerably lower spatial resolution than DSA, thus resulting in poor and limited visualization of small vessels. In the case of hypoplastic but present PcomA, CTA may not aid in differentiating ID from an aneurysm, particularly at the ICA-PComA junction. According to Min et al., the sensitivity, specificity, and accuracy of CTA in differentiating cerebral aneurysms from IDs remain considerably high (28). Magnetic resonance imaging techniques with time-of-flight and volume rendering have been reported to have similarly high sensitivity and accuracy in detecting cerebral IDs (29). Furthermore, geometric parameters on magnetic resonance angiography axial source images can provide added value in the diagnostic process. Magnetic resonance imaging enables easy visualization of silhouettes of tiny or hypoplastic vessels adjacent to the infundibulum with fast spin-echo and fast imaging by using steady-state acquisition sequences. With fusion imaging, Satoh et al. have viewed the outer wall configuration of the ipsilateral ICA-PcomA junction to distinguish and differentiate both lesions successfully (30).

Despite being promoted as the “gold standard,” DSA has several diagnostic and technical limitations in flow alterations and poor contrast filling of the PcomA during cerebrovascular catheter examinations. Recent technological improvements and software implementations with sophisticated smoothing and visualization algorithms have indicated the value of 3DRA, whose superior diagnostic properties to those of two-dimensional DSA imaging for evaluating cerebral IDs have been described (31).

Anecdotally, differentiating an aneurysm from an ID might not be the only dilemma faced by physicians. Labeling a “negative” radiological result as SAH in cases in which cerebral ID is present might be an equally difficult decision. The incidence of “normal” radiological findings in patients with SAH has been frequently reported in the literature. However, case reports are providing increasing evidence suggesting that these entities are not necessarily benign in nature (1, 29, 32–35). Particular radiological patterns, such as predominantly distributed SAH close to the observed infundibulum, may suggest the rupture site and the cause of the hemorrhage. For example, Yu et al. have found concentrated blood clots on CT located across the ipsilateral suprasellar cistern, interhemispheric cistern, and ipsilateral Sylvian fissure (33). Post-mortem reports have confirmed diagnoses of SAH associated with ID ruptures, thus underscoring the diagnostic challenges. Therefore, the question remains as to how often cerebral ID related hemorrhages are being neglected and misdiagnosed.

No defined guidelines or established clinical protocols exist regarding radiological follow-up of IDs in young patients. Because arterial IDs appear to be active lesions, radiological surveillance might be justified in these patients. In the literature, isolated case reports and case series have described progressive aneurysmal formation from ID (21, 26, 36, 37). Recently, Lee et al. have reported seven patients with ruptured PcomA IDs: two patients experienced directly ruptured IDs, whereas the other five experienced ruptured aneurysms originating from IDs. Only IDs of the PcomA had been incidentally identified during previous aneurysm treatment. However, the mean time between the identification of a PcomA ID and the rupture event was 9.2 ± 4.8 years. Fischer et al. have documented the objective transformation of a PcomA ID to a saccular aneurysm during follow-up angiography 7 years after the initial identification of the conical dilatation (38). Donauer et al. (39) have described a tiny acutely ruptured aneurysm (<2 mm fundus diameter) located on the infundibulum surface in a 62-year-old woman—an exemplary case demonstrating the feasibility and limitations of microvascular and endovascular approaches in managing these lesions.

In the present study, we reported our experience in diagnostic and endovascular management of ruptured cerebral IDs. Over more than 9 years, we identified eight patients with direct rupture of a cerebral ID. Multimodality imaging and specific radiological patterns drawn from the available literature allowed us to carefully conclude that an ID was the cause of the SAH in each case. In our experience, 3DRA remains the best diagnostic tool to successfully resolve any diagnostic discrepancies. Using post-processing reconstructions, we identified 0.5–0.7 mm blebs suspected to be ruptured sites. We strongly recommend performing at least one delayed radiological examination before planning any further action. Careful neuroprotection and blood pressure monitoring can meanwhile be successfully maintained.

Our experience in managing acutely ruptured complex and wide-necked aneurysms allowed us to successfully address these lesions. We consider endoluminal flow diversion to be an excellent stand-alone treatment candidate with high efficacy and a good safety profile (40). We acknowledge that acute SAH poses specific challenges to the safe use of FDs. Nevertheless, we suggest that careful analyses and strict selection of patients who could benefit from this integrated treatment are critical for its success.

It is essential to highlight that this study builds up around the SAH hypothesis due to ruptured IDs. The study case examples could only demonstrate that rupture can occur from an ID, but the inherent limitations of the design and the small sample size are not enough to the true nature of these lesions. Last but not least, with the more advanced FD technology, the available arsenal of stents, and the increased operator experience, we prove that such entities could be successfully adressed with a good safety profile. Tailoring a patient-specific approach with preoperative planning, adequate platelet inhibition and PRU testing, and competent operator experience should yield successful treatment results.

## Data Availability Statement

The raw data supporting the conclusions of this article will be made available by the authors, without undue reservation.

## Ethics Statement

Ethical review and approval was not required for the study on human participants in accordance with the local legislation and institutional requirements. The patients/participants provided their written informed consent to participate in this study.

## Author Contributions

SM, KS, KC, MP, and DM creating and writing the manuscript. MK, KM, KN, and VK participating in cases and review of the manuscript. SS performing the cases and final review. All authors contributed to the article and approved the submitted version.

## Conflict of Interest

The authors declare that the research was conducted in the absence of any commercial or financial relationships that could be construed as a potential conflict of interest.

## Publisher's Note

All claims expressed in this article are solely those of the authors and do not necessarily represent those of their affiliated organizations, or those of the publisher, the editors and the reviewers. Any product that may be evaluated in this article, or claim that may be made by its manufacturer, is not guaranteed or endorsed by the publisher.
